# Evolutionary History of the Risk of SNPs for Diffuse-Type Gastric Cancer in the Japanese Population

**DOI:** 10.3390/genes11070775

**Published:** 2020-07-10

**Authors:** Risa L. Iwasaki, Koji Ishiya, Hideaki Kanzawa-Kiriyama, Yosuke Kawai, Jun Gojobori, Yoko Satta

**Affiliations:** 1 Department of Evolutionary Studies of Biosystems, SOKENDAI (The Graduate University for Advanced Studies), Kanagawa 240-0193, Japan; iwasaki_risa@soken.ac.jp (R.L.I.); gojobori_jun@soken.ac.jp (J.G.); 2 Bioproduction Research Institute, National Institute of Advanced Industrial Science and Technology (AIST), Sapporo 062-8517, Japan; koji.ishiya@aist.go.jp; 3 Department of Anthropology, National Museum of Nature and Science, Ibaraki 305-0005, Japan; hkanzawa@kahaku.go.jp; 4 Genome Medical Science Project, National Center for Global Health and Medicine, Tokyo 162-8655, Japan; ykawai@ri.ncgm.go.jp

**Keywords:** genetic differentiation, PSCA, diffuse-type gastric cancer, rs2294008, Japanese, dual-structure model, Jomon people, Yayoi people, soft sweep, hardening selective sweep, selection target change

## Abstract

A genome wide association study reported that the T allele of rs2294008 in a cancer-related gene, *PSCA*, is a risk allele for diffuse-type gastric cancer. This allele has the highest frequency (0.63) in Japanese in Tokyo (JPT) among 26 populations in the 1000 Genomes Project database. *F_ST_* ≈ 0.26 at this single nucleotide polymorphism is one of the highest between JPT and the genetically close Han Chinese in Beijing (CHB). To understand the evolutionary history of the alleles in *PSCA*, we addressed: (i) whether the C non-risk allele at rs2294008 is under positive selection, and (ii) why the mainland Japanese population has a higher T allele frequency than other populations. We found that haplotypes harboring the C allele are composed of two subhaplotypes. We detected that positive selection on both subhaplotypes has occurred in the East Asian lineage. However, the selection on one of the subhaplotypes in JPT seems to have been relaxed or ceased after divergence from the continental population; this may have caused the elevation of T allele frequency. Based on simulations under the dual structure model (a specific demography for the Japanese) and phylogenetic analysis with ancient DNA, the T allele at rs2294008 might have had high frequency in the Jomon people (one of the ancestral populations of the modern Japanese); this may explain the high T allele frequency in the extant Japanese.

## 1. Introduction

Gastric cancer (GC) has a high incidence rate in East Asia and is the third leading cause of cancer death in the world [1]. GC is classified into two types, the diffuse type and the intestinal type [2]. The diffuse type of GC (DGC) is reported as geographically uniformly distributed [3] and the incident rate of DGC has been increasing compared with the intestinal type of GC [4,5]. A previous genome-wide association study identified *PSCA* (prostate stem cell antigen) on chromosome 8 as a susceptible gene for DGC in the Japanese [6]. The study analyzed 925 DGC cases with 1396 controls and reported a strong association with the T allele at rs2294008 in *PSCA*. This association has also been observed in other populations across the world including Koreans [7,8], Uzbekistanis [9], and even Caucasians [10,11,12].

rs2294008 is located in the upstream region of *PSCA,* and a T to C transition at this single nucleotide polymorphism (SNP) causes a missense mutation (ATG to ACG) at the presumed translation-initiating codon in the first exon, resulting in a nine-amino-acid truncation of its signal peptide. Tanikawa et al. [13] showed that the T/C variation caused differences in the subcellular localization and stability of PSCA proteins. The longer PSCA containing the risk (T) allele is presumed to be involved in cell proliferation as a signal protein, whereas short PSCA including the non-risk (C) allele downregulates PSCA expression with its protein degradation and subsequent activation of immune responses by antigen presentation [13]. They suggested that individuals with the T allele might have higher risk of GC, including DGC, as a result of accelerated cell proliferation due to suppressed PSCA protein degradation. Additionally, RT-PCR and immunohistochemical analysis revealed that the PSCA expression level is reduced in DGC cells [6]. Luciferase reporter assays also revealed that the T allele of rs2294008 reduced the transcriptional activity of the *PSCA* gene [6]. These findings support the conclusion that the T allele at the rs2294008 SNP can modulate PSCA transcriptional activity and protein functionality.

The frequency of this risk allele of DGC is the highest (0.630) in the Japanese in Tokyo (JPT) among the 26 worldwide populations in the 1000 Genomes Project (1KGP) [14]. Notably, other populations in East Asia showed lower frequencies of the T allele than that in JPT; e.g., 0.248 in Han Chinese in Beijing (CHB) [14], 0.260 in Taiwanese [15], and 0.497 in Koreans [16]. However, it is not clear why the frequency of this risk T allele has been maintained at high frequency only in JPT. In the present study, we explore the evolutionary forces (genetic drift or natural selection) that may be responsible for this high frequency in JPT through: (i) the examination of the signature of natural selection operating on either the T or C alleles at the rs2294008 SNP, (ii) exploring the evolutionary trajectory of the risk allele through computer simulations, and (iii) a phylogenic analysis of ancient genome sequence data from one of the ancestral populations of the extant Japanese population, the Jomon people.

## 2. Materials and Methods

### 2.1. Strategy of Analysis

First, using the *F*_ST_ value between JPT and CHB, we evaluated the extent of T allele frequency difference at rs2294008 compared with genome-wide SNPs. Then, we examined whether the large allele frequency difference could be explained by positive selection on either allele using multiple neutrality tests. We detected a signature of positive selection on the C allele in CHB and then addressed why the non-positively selected T allele could have been maintained at a high frequency in JPT. We tested whether demographic events of JPT could attain the high *F*_ST_ value at rs2294008 using allele frequency simulation. Moreover, to confirm this demographic effect, using ancient genome sequences of the Jomon people, we investigated their phylogenetic relationship with the extant JPT/CHB.

### 2.2. Human SNP Data

We retrieved variant call format (VCF) data from two local populations (JPT, CHB) and four metapopulations (East Asian excluding JPT and CHB (non JPT/CHB EAS); European (EUR); South Asian (SAS); African (AFR)) from 1KGP Phase 3 [14] and used the GRCh37 genomic positions (or coordinates) for each SNP. The ancestral/derived alleles are defined in 1KGP [14].

### 2.3. F_ST_

We estimated differentiation level by Hudson’s *F*_ST_ [17,18] and examined the relative extent of differentiation at rs2294008 among genome-wide SNPs between JPT and other East Asian populations such as CHB (Table S1). For this calculation, we removed indels, coordinated/overlapped SNPs, and multi-allelic SNPs. The *MHC* region (chr6: 25,726,291-33,368,333) was also removed because it contained many genes governed by different evolutionary mechanisms such as balancing selection [19]. We drew a Manhattan plot based on the *F*_ST_ values across the entire genome using the program qqman [20].

### 2.4. Linkage Disequilibrium Analysis

Linkage disequilibrium (LD) between rs2294008 and other SNPs in flanking regions was examined by D’ [21]. In this calculation, SNPs with minor allele frequency (MAF) < 0.05 were removed and the LD block was determined with the software Haploview (v 4.2) [22] using the criterion of D’ > 0.98 [23].

### 2.5. Neutrality Tests

#### 2.5.1. Haplotype-Based Tests (EHH, nS_L_, and H12)

We performed three haplotype-based tests, extended haplotype homozygosity (EHH), number of segregating sites by length (*nS*_L_), and haplotype homozygosity (H12), for detecting recent or ongoing positive selection on rs2294008 [24,25,26]. We calculated the EHH for a 400 kb region (chr8: 143,563,622-143,963,622) surrounding rs2294008. Both alleles at rs2294008 (T/C) were used as a single core site for the EHH test. SNPs with MAF < 0.05 were removed in the test. The *nS*_L_ (implemented in selscan [27] (v 1.1.0)) was measured as haplotype length linked with a derived C allele based on the number of segregating sites in a haplotype, and not based on a recombination map. We used 303,438 and 377,335 bi-allelic SNPs with MAF > 0.01 on chromosome 8 in JPT and CHB, respectively. We set three maximum window sizes for this analysis: 100 SNPs (default), 1500 SNPs, and no size limitation. H12 detects hard and soft sweeps with homozygosity based on the frequency of the two most common haplotypes [26]. We scanned chromosome 8 using sliding windows of 125 SNPs (for JPT) or 124 SNPs (for CHB), which were equivalent to the number of SNPs in the 21.9 kb LD block (chr8: 143,752,235–143,774,193) in each population, and then calculated H12.

#### 2.5.2. Site Frequency Spectrum-Based Tests (Tajima’s D and Fay and Wu’s H)

To investigate the signatures of natural selection across the 21.9 kb LD block, we also performed two site frequency spectrum-based tests, Tajima’s *D* [28] and normalized Fay and Wu’s *H* [29,30], and calculated p-values using DnaSP (v 6.0) [31]. All gaps and sites for which the ancestral alleles are unknown were excluded from the analysis.

#### 2.5.3. Nucleotide Diversity (π)

To evaluate the reduction of genetic diversity due to putative selective sweep, we examined three regions and, for each region, calculated the nucleotide diversity [32] π_C_ [31] of C haplotypes or π_T_ of T haplotypes in JPT and CHB separately using DnaSP. The three regions were: (i) the 21.9 kb LD block (chr8: 143,752,235–143,774,193), and its (ii) upstream (chr8: 143,652,235–143,752,234) and (iii) downstream 100 kb flanking regions (chr8: 143,774,194–143,874,193). The number of C haplotype sequences in JPT and CHB was 77 and 155, respectively, and that of T haplotypes was 131 and 49, respectively. In order to avoid the overestimation of a π value caused by the inclusion of a recombinant between C and T haplotypes, we removed two possible recombined haplotypes in CHB. We could not detect any recombined haplotypes in JPT. We then used z tests to compare the π_T_ or π_C_ values of the 21.9 kb LD region between JPT and CHB as well as the π_T_ or π_C_ values of the LD region with that of flanking 100 kb regions within each population. For the application of false discovery rate (FDR), we corrected the *p*-values of each summary statistic into *q*-values with the Benjamini–Hochberg procedure [33]. Furthermore, we compared the nucleotide diversity of two subhaplotypes, C-A and A-G (see below for the definition of subhaplotypes).

#### 2.5.4. Two-Dimensional Site Frequency Spectrum (2D SFS)

We evaluated intra allelic variability (IAV) within a derived allele (D) group using 2D SFS (Φ_i,j_ [34]) and its summary statistics, F_c_, G_c0_, and L_c0_ [34,35]. The latter three summary statistics were designed to detect incomplete selective sweep signals when compared to the null distribution of each summary statistic. Each null distribution was generated under a neutral model using ms [36] with at least 1000 replications under the same demographic parameters used previously [35,37]. For the application of FDR, we applied the Benjamini–Hochberg procedure to the 2D SFS statistics [33]. We judged cases as presenting ongoing positive selection if at least two summary statistics were statistically significant (*q*-value < 0.05). We tested for selective sweep with rs2294008 in a core region of chr8: 143,755,915–143,770,914 (15 kb) or chr8: 143,755,876–143,771,875 (16 kb) in JPT and CHB, respectively. Each core region was determined by *r*^2^ > 0.75 with rs2294008, following prior criteria [34]. In addition, we evaluated the IAV of two subhaplotypes of C within a population. A subhaplotype was defined as the combination of alleles at two sites (rs2976391 (C/A) and rs2978983 (A/G)) linked with C at rs2249008, and named C-A and A-G subhaplotypes, respectively. We evaluated the lower limit of the start of positive selection for the two subhaplotypes, using a mutation rate of 0.5 × 10^−9^/site /year [34].

#### 2.5.5. Application of Population Branch Statistics (PBS)

We examined the signal of local adaptation acting on the C allele in CHB and JPT using PBS analysis, which is one of the *F*_ST_-based summary statistics. We selected sites for calculating *F*_ST_ following previously described criteria [38]. Then, using EUR as an outgroup population, we calculated Hudson’s *F*_ST_ per SNP site pairwise among JPT, CHB, and EUR. We calculated PBS values for genome-wide SNPs of JPT and CHB following previous methods [38,39]. Then, we compared the ranking of PBS of rs2294008 in JPT and that in CHB.

### 2.6. Forward Simulation Using Japanese Demographic Model

We performed forward simulation of allele frequency trajectory at the SNP (T/C) under the Wright–Fisher model [40] of haploidy. This simulation analysis has two conditions under a demographic model (see below, Figure S1): (i) both T and C alleles are neutral, and (ii) the C allele is positively selected only in simulated CHB lineages.

#### 2.6.1. Demographic Parameters in Simulations

The demographic “dual structure model” [41] proposes that the extant genetic and phenotypic diversity in the Japanese populations were caused by the past admixture of two genetically different populations, the Jomon and immigrant Yayoi farmers. The Jomon people are indigenous and inhabited the Japanese archipelago from at least 16,000 years ago (ya) [42], whereas the immigrant Yayoi farmers originated from the Asian continent [43] and migrated to the Japanese archipelago 2500 ya [42] and then admixed with the Jomon people. This model has been supported by previous studies based on ancient or extant genomic data from the Japanese [44,45]. 

From a common ancestor, the simulated Jomon people (JMN) and simulated ancestral population of the Asian continent (A_CNT) diverged and were isolated for *t1* generations. After this separation, these two populations admixed with a rate *r* of the JMN component. After *t2* generations of admixture, we calculated Hudson’s *F*_ST_ [17] between simulated JPT, who are descendants of the admixed population, and simulated CHB, who are those of A_CNT. No migration between simulated JPT and simulated CHB was assumed throughout the simulation based on previous studies [43,44,45,46]. 

In the above simulation, we used four variable parameters and three constants. The first parameter was *t1*, and we used five *t1* values (875, 1125, 1375, 1875, and 2375 generations), corresponding to 17,500 to 47,500 years [42,44]. Generation time is assumed to be 20~30 years [47], and we used a shorter generation time to be conservative regarding allele frequency changes in the simulations. The second parameter was *r* for the admixture proportion of JMN; we used three values: 0.4, 0.2, and 0.1 [43,44,45,46]. The third and fourth parameters were the population size for JMN (*N*_JMN_) and A_CNT (*N*_A_CNT_), respectively. For *N*_JMN_, we used 500, 1000, 2000, 4000, 8000, 10,000, 12,500, and 15,000, whereas for *N*_A_CNT_, we used 1000, 2000, 4000, 8000, 16,000, 25,000, and 30,000. We added the condition that *N*_JMN_ was always equal to or smaller than *N*_A_CNT_. In contrast, three constants were used: *t2* = 125 generations [42], *N*_simJPT_ = 12,824 and *N*_simCHB_ = 29,204 [44]. The number of combinations of these parameters was 570 and each combination was simulated 10,000 times for each initial frequency of T allele (f_i_) in the ancestral population, ranging from 0.1 to 0.9 with increments of 0.1. For simulations incorporating selection, we used one additional parameter, the selection coefficient (*s*). We used 2 × *N*_simCHB_ × *s* = 1, 10, 50, or 100. Selection occurred on the CHB lineage for 126 generations (the time at and after admixture between A_CNT and JMN). 

#### 2.6.2. Investigating Possible Causes of Large *F_ST_*

For each parameter combination, we drew a histogram for 90,000 simulated *F*_ST_ values under neutrality. These 90,000 *F*_ST_ values did not include cases where alleles T or C were fixed in both JMN and A_CNT before admixture or fixed in both the simulated JPT and CHB after admixture. Negative *F*_ST_ was regarded as zero. The simulated *F*_ST_ histogram was compared with the empirical *F*_ST_ data. To do so, we needed a proportion (p_i_) of nine f_i_ in the common ancestor of JMN and A_CNT. Each *F*_ST_ was weighted by the estimated p_i_ of the empirical derived allele frequency. p_i_ was calculated from 570,129 SNPs on chromosome 8 in JPT as p_i_ = 0.5589, 0.0984, 0.0734, 0.0623, 0.0494, 0.0436, 0.0360, 0.0342, and 0.0438 (i = 1~9). 

We then examined whether high T allele frequency and high *F*_ST_ of a particular SNP are simulated under a neutral state. Among 570 combinations, 410 showed simulated *F*_ST_ distributions that were not significantly different from the empirical one based on the two-sample Kolmogorov–Smirnov test (*p*-value ≥ 0.05) implemented in the Python package scipy (v. 0.19.1) [48]. Then, of these 410 combinations, we further chose combinations in which there was a SNP with a higher *F*_ST_ and higher T allele frequency than the observed values. We also checked whether these combinations fulfilled the condition that T allele frequency must be higher in JPT than CHB but that neither alleles are fixed in either population. Additionally, we examined cases of positive selection acting on the C allele in the simulated CHB lineage. We reused 410 combinations that could reproduce similar *F*_ST_ distributions under neutrality and further simulated a total of 1640 combinations (four selection coefficients on the C allele, each for 410 combinations). Then, we counted the number of cases which attained high *F*_ST_ (>0.2547) and high T allele frequency (>0.62) in simulations under positive selection or neutrality.

### 2.7. Analysis using Ancient DNA Sequences from the Jomon People

In order to examine whether the extant T haplotype in JPT was derived from those in the Jomon, we used three reported ancient individual genomes of the Jomon people, the Ikawazu (IK002) [49] and two Funadomari (FUN23, FUN5) [46]. We then constructed a median-joining haplotype network together with the extant JPT and CHB sequences using network (v. 5.0.1.1) [50,51]. A chimpanzee orthologous sequence (Pan tro 3.0:8:145391694:145412716), obtained using the extant human sequence of 21.9 kb LD region as a query by nucleotide blast, was used as an outgroup. We chose reliable sites from the Ikawazu and Funadomari Jomon sequences by the following method. Unreliable sites in IK002 were filtered out with the HaplotypeCaller algorithm implemented in GATK (v. 3.8) with gVCF mode (sites with mapping quality < 20 or base quality < 10). Further, we removed sites which were likely to be derived from sequence error and checked the IK002 coverage of each site. We then produced a consensus individual sequence when the coverage of the genome sequence was low [49,52]. We chose sites with depth > 2 and genotype quality > 1. At a bi-allelic site, the allele which had deeper coverage than the other was chosen. The genomes of both samples of Funadomari were phased according to the previous method [46]. Unreliable sites were filtered out with the HaplotypeCaller algorithm implemented in GATK with gVCF mode and were filtered with the variant quality score recalibration (VQSR) approach. All sites examined in this study were restricted to sites listed in 1KGP-Phase 3 SNPs [6]. Furthermore, we chose sites with genotype quality > 30 and depth > 30 for high coverage sample FUN23 and genotype quality > 20 for the low coverage sample FUN5. FUN23 was treated as a diploid sample but FUN5 was treated as a haploid sequence in the same way as IK002 due to low coverage. Singleton sites which were unique in the three ancient samples (IK002, FUN23, and FUN5) were determined under the infinite site assumption [46] and were removed from sequences.

## 3. Results

### 3.1. The T Allele at rs2294008 is Highly Differentiated between JPT and CHB

**Figure 1:**
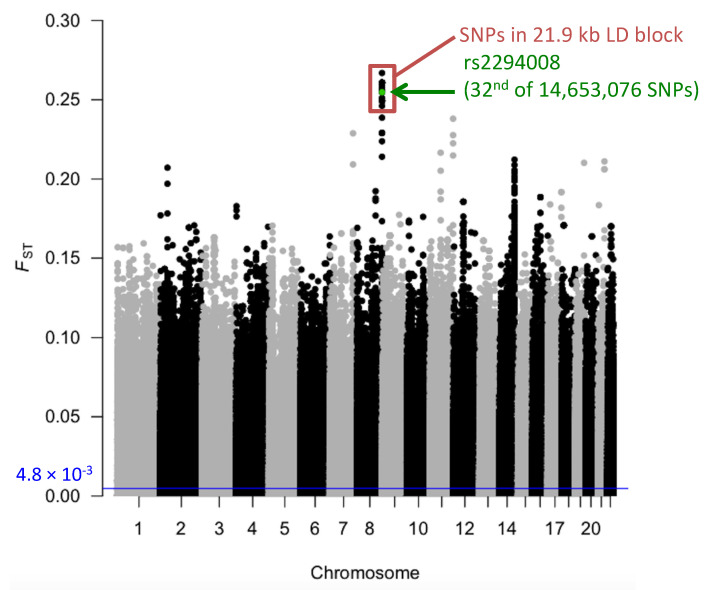
Manhattan plot of *F*_ST_ values between JPT and CHB. Each dot represents an *F*_ST_ value of each autosomal SNP. Green dot represents the *F*_ST_ value at rs2294008 and red box indicates SNPs in the LD block (21.9 kb) containing rs2294008. The average *F*_ST_ (4.8 × 10^−3^) is represented by the blue horizontal line.

**Figure 2:**
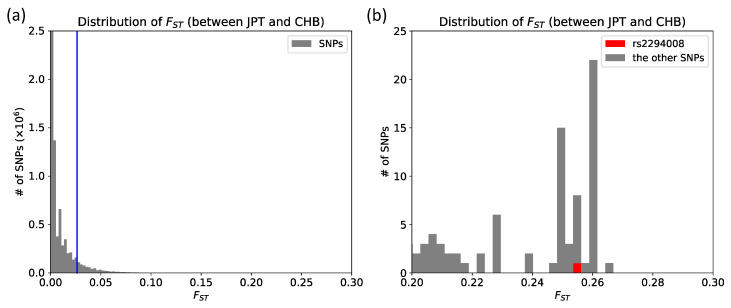
Empirical *F*_ST_ distribution. (**a**) *F*_ST_ distribution of the entire genome between JPT and CHB. The blue line represents the fifth percentile of distribution (*F*_ST_ ≥ 0.0264); (**b**) Local zoom (0.20 < *F*_ST_ < 0.30) of (**a**). rs2294008 is marked by a red rectangle among 14,653,076 SNPs in the entire genome.

JPT has high frequency (0.63) of the risk T allele at rs2294008 for DGC (Table S1). We compared the extent of genetic differentiation, *F*_ST_, of rs2294008 to all other genomic SNPs between JPT and CHB (total of 14,653,076 SNPs), and found that the *F*_ST_ of rs2294008 is significantly high at the genome level (*F*_ST_= 0.2547, Figure 1, Figure 2, and Table S2). 

**Figure 3:**
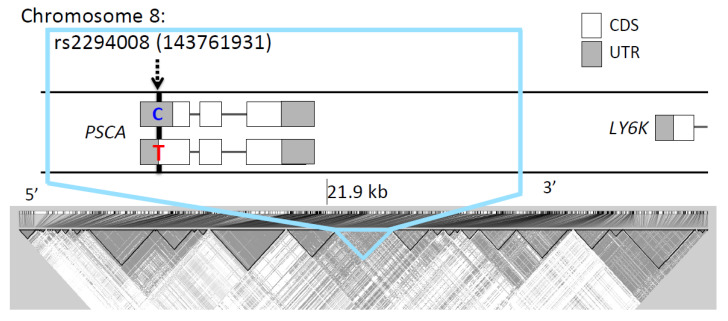
Genomic context (upper) and linkage disequilibrium (lower) of rs2294008. The gray and white squares represent coding sequence (CDS) and untranslated region (UTR) of a gene, respectively. rs2294008 (T/C) is located in a PSCA initiation codon (chr8: 143,761,931). Sequences with the C allele are truncated by nine amino acids compared with sequences with the T allele [13].

The rs2294008 SNP was located in a 21.9 kb LD block (chr8: 143,752,235-143,774,193, D’ > 0.98) (Figure 3) that was comprised of 125 SNPs in JPT. Most of the top 50 SNPs (49/50) of genome-wide *F*_ST_ values are located in this LD block (Table S2). We compared the *F*_ST_ for this block with other blocks of the same size (21.9 kb) on chromosome 8. The block containing rs2294008 showed the highest *F*_ST_ value among 6,510 blocks (*F*_ST_ = 9.8 × 10^−2^, Figure S2). Therefore, we concluded that rs2294008 and its tightly linked SNPs were significantly differentiated between JPT and CHB.

### 3.2. Exploring the Signal of Natural Selection Acting on rs2294008

**Figure 4:**
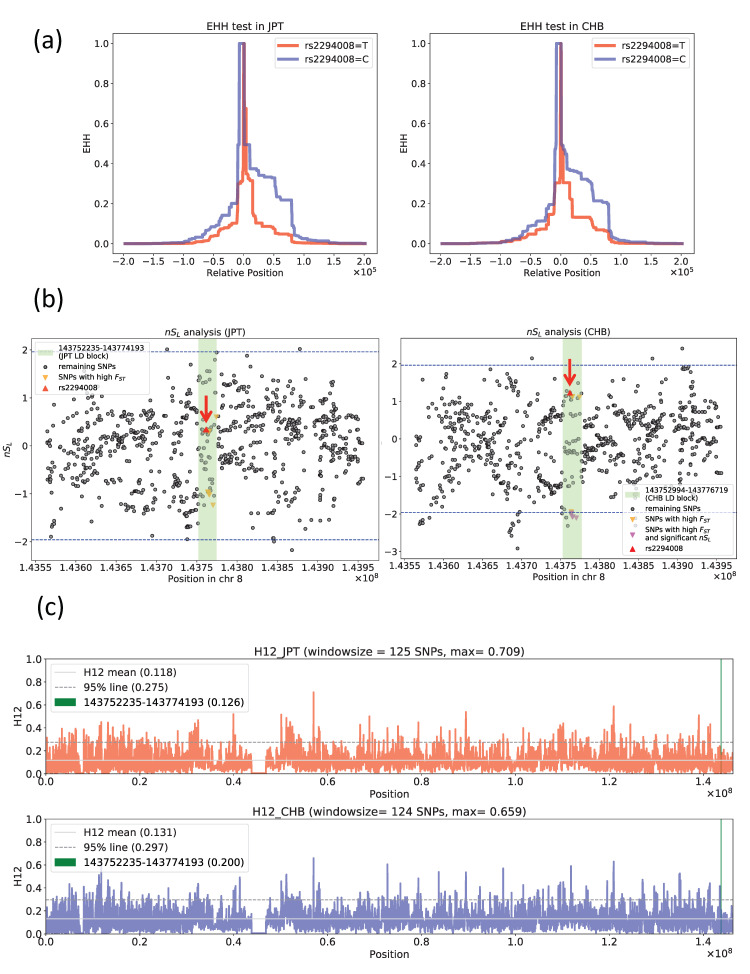
Neutrality tests (Extended haplotype homozygosity (EHH), number of segregating sites by length (*nS*_L_) and haplotype homozygosity (H12)). (**a**) EHH of the region surrounding rs2294008 in JPT (left) and CHB (right). (**b**) *nS*_L_ of the region surrounding rs2294008 in JPT (left) and CHB (right). There was no limitation on the maximum size of the window. Each *nS*_L_ value at rs2294008 in CHB and JPT is indicated by a red triangle, while the top ten highly differentiated SNPs in CHB are indicated by inverted magenta triangles and the corresponding values in JPT are indicated by orange inverted triangles. The light green belt indicates the LD block region containing rs2294008 (JPT: 143,752,235-143,774,193, CHB: 143,752,994-143,776,719). Blue dashed lines represent 95% confidence intervals. (**c**) Sliding window analysis of H12 surrounding rs2294008 in JPT (upper) and CHB (lower). Light gray solid lines represent the H12 mean values within each population. The gray dashed lines represent the lower 95% line of H12 within each population. The light green line represents the JPT LD block region (143,752,235-143,774,193) used in this analysis. Values in parentheses in labels represent each H12 value.

We found that rs2294008 was one of the most differentiated SNPs between JPT and CHB. This highly differentiated SNP in JPT may be explained by natural selection on the C allele in CHB and/or natural selection on the T allele in JPT. To examine the signature of natural selection, two different types of summary statistics were applied: (i) haplotype-based statistics (EHH, *nS*_L_, and H12) and (ii) site frequency spectrum-based statistics (Tajima’s *D*, normalized Fay and Wu’s *H*, and PBS). EHH showed no clear signal of selective sweep acting on the T allele at rs2294008 in JPT and CHB (Figure 4a). Moreover, no signal of selective sweep on the C allele was detected in CHB or in JPT (Figure 4a). Similarly, none of the five SNPs with high *F*_ST_ values (0.2608~0.2669) in the same LD region showed clear signals in either population (Figure S3). Additionally, neither *nS*_L_ nor H12 showed any significant signals of hard/soft sweeps on rs2294008 in both JPT and CHB (Figure 4b,c). *nS*_L_ of rs2294008 showed an insignificant value, 0.4063 (*p* > 0.05) (Figure 4b). Six of the ten SNPs with high *F*_ST_ in the 21.9 kb LD block showed a marginally negative *nS*_L_ value in CHB (*nS*_L_ = −1.978 ~ −2.106; 0.01 < *p* < 0.05) (Figure 4b), but the signal for a haplotype containing these SNPs was not clear based on H12 sliding window analysis (H12 = 0.118 (*p* > 0.05) and 0.126 (*p* > 0.05) for JPT and CHB, respectively).

In the 21.9 kb LD block region including rs2294008, Tajima’s *D* showed no clear signature of natural selection (Tajima’s *D* = 2.031 (0.10 > *p* > 0.05) and 0.745 (*p* > 0.10) for JPT and CHB, respectively). In contrast, normalized Fay and Wu’s *H* detected a weak signal of an excess of high-frequency-derived alleles in CHB (normalized Fay and Wu’s *H* = −0.319 (*p* > 0.10) and −2.174 (0.01 < *p* < 0.05) for JPT and CHB, respectively) due to selection or demographic effects, e.g., recent bottleneck or metapopulation structure [53,54]. PBS analysis did not detect any local adaptation signal in JPT or CHB (rs2294008: PBS value = 0.0592 and 0.0685, ranked 23,975th and 11,542th of a total 9,051,837 SNPs, respectively (Figure S4)). These observations suggest that there is no clear signature of positive selection on rs2294008 in either population.

### 3.3. Two-Dimensional Site Frequency Spectrum (2D SFS)

The newly developed 2D SFS statistics F_c_, L_c0_, and G_c0_ [34,35] were applied to explore a signature of selective sweep on the region containing rs2294008. These summary statistics detected low IAV among sites linked with a putative targeting derived allele (C allele at rs2294008) due to incomplete selective sweep.

#### 3.3.1. Detection of Positive Selection in CHB, but not in JPT

Signatures of selective sweep with the C allele at rs2294008 in both JPT and CHB were examined using 2D SFS statistics. We detected a significant signature in CHB using all three summary statistics (Table 1). Statistics for the C allele were significantly lower than those for neutrality and the T allele. This reveals a significant reduction of IAV for the C allele in CHB and suggests that this allele is very likely to have been positively selected. Conversely, JPT did not show any signature of positive selection acting on either allele (Table 1).

#### 3.3.2. Selection Mode in CHB and JPT

We examined whether the positive selection signal on the C allele was caused by classic hard sweep or soft sweep. In this study, we followed the definition of soft sweep whereby more than one distinct selection-targeting haplotype was present in the D group [34]. The summary statistics (G*_c0_ and γ*(10), *i*_max_) for CHB fulfilled the criteria for hard sweep [34]. However, a large value of i*_max_ of CHB (i*_max_ = 75) indicated that the D group was classified into several large groups of haplotypes (subhaplotypes). In fact, the two subhaplotypes, classified by two sites (rs2976391 and rs2978983), could be the target of positive selection. We found that 75 chromosomes had derived alleles and the remaining 80 chromosomes had ancestral alleles in the D group at the two sites; we named the former as the A-G subhaplotype and the latter as the C-A subhaplotype based on the allele combination at rs2976391 (C/A) and rs2978983 (A/G). 

We examined whether these subhaplotypes in CHB have a signal of positive selection using 2D SFS. Both subhaplotypes had a significant signal of positive selection identified by three summary statistics (Table S3). Then, we compared the π values between the two subhaplotypes in CHB and confirmed the effect of selective sweep. The two subhaplotypes in CHB showed quite similar genetic diversity (π_A-G_ = 0.4 × 10^−4^, π_C-A_ = 0.4 × 10^−4^, π_A-G vs C-A_ = 2.4 × 10^−4^). These observations were in good agreement with the results of 2D SFS.

These two subhaplotypes were also observed in JPT and we examined whether they were under positive selection in JPT. We detected a positive selection signature on the C-A subhaplotype, but not the A-G subhaplotype (Table S3). This suggests that within the alleles containing C at this SNP, the C-A subhaplotype under positive selection (hard sweep) may have been masked by the non-selected A-G subhaplotype, resulting in the failure to detect a signal of positive selection when the subhaplotypes were grouped. Taken together, our results support that JPT and CHB were under different selection modes.

#### 3.3.3. History of Natural Selection in JPT and CHB

The upper limit of time of positive selection on the C allele in CHB is equivalent to the divergence time of the C-A and A-G subhaplotypes. We estimated that positive selection on the C allele began at most 240,000 ya, estimated from the divergence between these two subhaplotypes. The lower limit of positive selection beginning on each subhaplotype in CHB was estimated with the time to most recent common ancestor (TMRCA) within each subhaplotype [34]. We estimated that selection occurred circa 30,000 ± 12,000 ya (C-A subhaplotype) or circa 27,000 ± 14,000 ya (A-G subhaplotype) in CHB. Selection on the C-A subhaplotype in JPT occurred circa 11,000 ± 8000 ya. In CHB, this ongoing selection on the two subhaplotypes in the extant population led to the high allele frequency of the rs2294008 C allele. In contrast, selection on the A-G subhaplotype in JPT had relaxed at some point. The selection coefficient of the C-A subhaplotype in JPT was not likely to be strong enough to allow the detection of a signature of classic selective sweep on all C alleles. Consequently, the C allele frequency is maintained at an intermediate range in JPT, which results in a large C allele frequency difference between the two populations. 

### 3.4. Examination of Whether Genetic Drift Can Explain the High F_ST_ Using Forward Simulation

**Figure 5:**
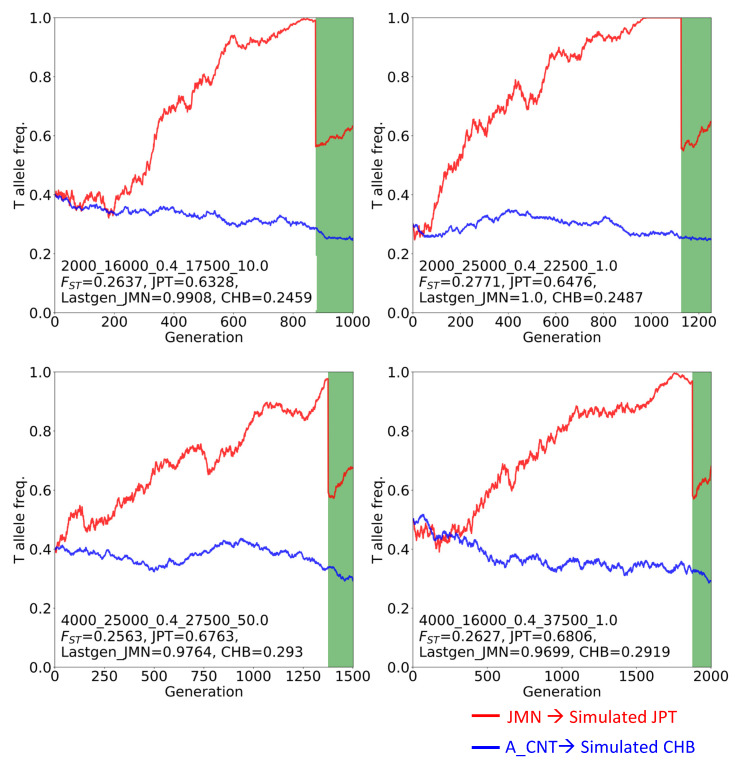
Four T allele frequency trajectories (out of 35) cases which satisfy the high *F*_ST_ and high T allele frequency in simulated JPT under positive selection on the C allele in the simulated CHB lineage. Red lines represent T allele frequencies of the simulated Jomon population (JMN) and simulated JPT. Blue lines represent those of the simulated ancestral population of the Asian continent (A_CNT) and simulated CHB. Green belts represent post-admixture of JMN and A_CNT. Subtitles display information on the parameters, *F*_ST_ value, and the T allele frequency of the simulated JPT, of the last generation in the JMN and of the simulated CHB. All 35 trajectories under positive selection and three trajectories under neutrality are shown in Figures S5 and S6, respectively.

The rs2294008 T allele is the major allele in JPT, even though one of the subhaplotypes with the C allele is likely to be under ongoing positive selection. Considering the possibility that Japanese population-specific demography caused this high allele frequency in JPT, we simulated the T allele frequency trajectory by performing forward simulation under neutrality or selection only in CHB. We constructed a demographic model for JPT and CHB following the “dual structure model” proposed by Hanihara [41] (Figure S1) and examined 570 parameter combinations of four parameters (*t1*, *N*_JMN_, *N*_A_CNT_, and *r*). Under the neutral state in CHB and JPT, we found three cases from three combinations that satisfy the high *F*_ST_ and high T allele frequency in simulated JPT (Figure S5). All three cases showed a similar pattern of allele frequency trajectory; the T allele frequencies at the last generation of JMN ranged from 0.94 to 1.0, and dropped by approximately half after admixture. These conditions lead to the actual allele frequency in JPT (approximately 0.6). The length of *t2* (2500 years, 125 generations) was too short to change allele frequency dramatically from those after admixture in the simulated JPT lineage. In other words, a large difference of allele frequencies between the two extant populations and high T allele frequency in the extant JPT would require high T allele frequency from the JMN lineage. We found that the highest *F*_ST_ values could be attained if the T allele was nearly fixed in one of the ancestral populations or nearly lost in the other population (Table S4). Additionally, we performed simulations under four selection coefficients on the C allele in the CHB lineage. Based on this, we expected to get a fourfold higher number of combinations/cases relative to that under the neutral state of three cases from three combinations; however, we found an exceedingly high number of cases (35 cases from 24 combinations) (Figure S5 and S6). We also found a high T allele frequency in JMN and high *F*_ST_ between simulated JPT and CHB (Figure 5, Figure S6, and Table S4). Taken together, the T allele frequency in the Jomon must have been higher than that in the continental immigrant population and so the extant JPT inherited the T allele from the Jomon people. This led to the empirically high *F*_ST_ of rs2294008 between JPT and CHB, even though the T allele is a risk allele of severe disease.

### 3.5. Phylogenic Position of Jomon Haplotypes in the Network of Extant JPT and CHB

We classified the haplotypes of extant JPT and CHB into 93 haplotypes (Table S5); 41 were JPT-specific, whereas 37 were CHB-specific and 15 were shared between JPT and CHB. Of the 41 haplotypes specifically found in JPT, 87.8% (36 haplotypes) were harboring the T allele at rs2294008 (i.e., T haplotypes). Conversely, 27.0% (10 haplotypes) of the 37 CHB-specific haplotypes were T haplotypes. This indicates that the number of T haplotypes specific to JPT was larger than that in CHB, suggesting that T haplotypes in JPT were maintained for a longer time than in CHB.

**Figure 6:**
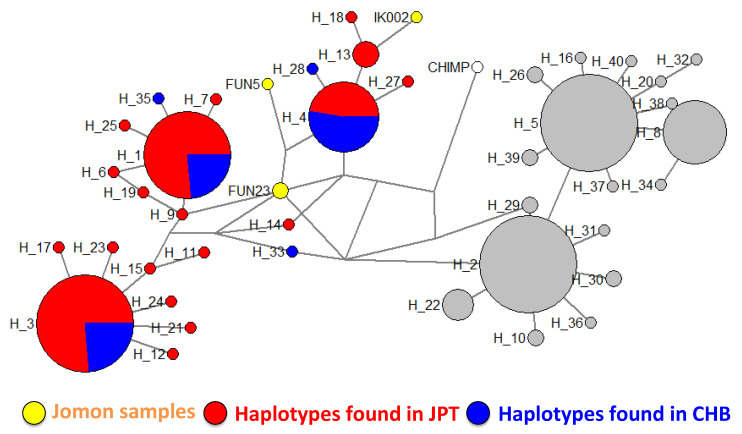
Network of haplotypes from three Jomon samples, extant JPT, extant CHB and the chimpanzee. Haplotypes which have C alleles are represented by gray circles and the other haplotypes are colored (Jomon samples (IK002, FUN5, and FUN23): yellow; extant JPT: red; extant CHB: blue; chimpanzee (CHIMP): white). The size of the circle represents the number of haplotypes. The branch length is not proportional to the number of substitutions. The labels starting with “H” represent the haplotype number defined in our network analysis. The corresponding haplotype numbers defined in extant samples are presented in Table S5.

Based on the simulation results, we hypothesized that the extant Japanese T allele was mainly inherited from the Jomon people. To evaluate this hypothesis, we compared sequences from the extant JPT to those from ancient genomes of Jomon samples, Ikawazu and Funadomari [46,49]. We found that all ancient genomes of Jomon samples had the T allele at rs2294008 as we expected. Then, we constructed a median-joining network and examined the relationship between extant JPT haplotypes and the Jomon haplotypes (Figure 6). The network formed two clusters, T haplotypes and C haplotypes, and all Jomon samples were clustered with the T haplotypes in JPT. Two Jomon samples, FUN23 (Funadomari) and IK002 (Ikawazu), were closely related to extant Japanese-specific haplotypes including haplotype 61 (H_9) for FUN23 and haplotype 30, 31, and 87 (H_13) for IK002. The haplotype of FUN5 was most closely related to that of FUN23 as well as four major haplotype groups in JPT and CHB (haplotypes 19, 42, 43, and 84 (H_4)). Sequences from the Funadomari Jomon were classified into different clusters in the network. FUN23 and FUN5 were reported to not share the same maternal lineage [46], suggesting that these two Jomon individuals likely had a low degree of kinship and that haplotypes had diverged within the population. Although IK002 and FUN5 have low coverage and the phylogenetic position of the haplotypes are uncertain, our results also support that some T haplotypes in the extant JPT are derived from Jomon haplotypes.

## 4. Discussion

A significant association between the T allele at rs2294008 and diffuse-type gastric cancer (DGC) was reported in the Japanese [6]. The risk allele (T) frequency in JPT was found to be higher than that in other East Asian populations in 1KGP (Table S1). Although this SNP showed high genetic differentiation (*F*_ST_) between JPT and CHB, most neutrality tests applied did not find any obvious signature of positive selection acting on this SNP or the region containing this SNP, except for 2D SFS (F_c_, G_c0_, and L_c0_). The 2D SFS tests are specific in detecting incomplete selective sweep based on IAV linked with a putative target site [34]. This method is robust to recombination rate variation (i.e., the existence of recombination hotspots), unlike other haplotype-based selection detection tests. For example, in our study, we examined ten SNPs with high *F*_ST_ values using the *nS*_L_ method (Figure 4b). Only six of the ten SNPs showed a signal of positive selection, although it was marginal. In fact, the distribution of *r*^2^ with rs2294008 showed an abrupt reduction of values outside the 15 kb region (where 2D SFS tests were applied) in JPT (Figure S7). The presence of a hotspot near the putative target (rs2294008 in the present study) would have a high chance of dissipating linkage disequilibrium unless positive selection occurred very recently. Since *nS*_L_ has been previously used to detect natural selection in the form of relatively recent soft sweep [25,35,55], both the presence of a hotspot and older soft sweep would lead to the failure to detect natural selection by *nS*_L_. This indicates that 2D SFS is more robust in detecting older soft sweeps than *nS*_L_ due to its robustness to recombination rate fluctuation.

When we examined the level of π in JPT and CHB (Table S6), we found that the π_C_ values of the 21.9 kb region (chr8: 143,752,235–143,774,193) between CHB and JPT were not significantly different from each other (1.6 × 10^−4^ and 1.5 × 10^−4^, respectively (*p* = 0.3967)). The π_C_ values of the 21.9 kb region in both populations were significantly lower than those of the adjacent 100 kb regions (all *q* < 0.01). In contrast, the π_T_ values of the 21.9 kb region showed marginal (for CHB) or significant (for JPT) reduction in the upstream flanking region (0.01 < *q* < 0.05 and *q* < 0.01, respectively), but they did not show significant reduction in downstream flanking regions in both populations (all *q* > 0.05). This suggests that the reduction of genetic diversity in the 21.9 kb LD block of the C allele was not caused by mutation rate fluctuation, but rather by a common evolutionary force that occurred in both populations. In other words, the reduction of π_C_ may have been caused by a common selection event shared between JPT and CHB. Even though only one subhaplotype was selected in JPT, π_C_ could reflect the operation of natural selection and consequently display a small π_C_ value. Taken together, the comparison of π_C_ supported the results from 2D SFS that positive selection operated on one subhaplotype in JPT, and both subhaplotypes in CHB.

The application of 2D SFS clearly indicated a signature of soft sweep in CHB but not in JPT (Table 1). Although the allele configuration between JPT and CHB is very similar, this discrepancy is attributed to a difference in selection mode and selection targets between the two populations. Both the C-A and A-G subhaplotypes were targets of ongoing positive selection in CHB at least 30,000 ya and 27,000 ya, respectively, which may have led to high C allele frequency in CHB. This time frame is consistent with the divergence between the Jomon people and the continental Asian population (15,000~38,000 ya [44,45,46]). Conversely, in JPT, only the C-A subhaplotype is a target of ongoing positive selection (Table S3) and selection in JPT begun almost simultaneously with that in CHB. These observations suggest that both subhaplotypes were targets of positive selection in the common ancestor of JPT and CHB. However, positive selection on the A-G subhaplotype in JPT was relaxed/ceased at some time point in the Jomon lineage and the selection mode observed in JPT was the hardening of soft selective sweep with the C-A subhaplotype. This suggests that positive selection on the C-A subhaplotype occurred in both JPT and CHB, and was not a result of local adaptation. PBS analysis specifies that unique local adaptation in a specific population is based on the long branch of *F*_ST_ [38]. If ongoing positive selection had operated in two populations, a long branch would not be limited to a specific population and would lead to less power of detection. Therefore, this caused the failure to detect positive selection in CHB/JPT using PBS analysis. rs2976391 (C/A), one of the two SNPs which defined the two subhaplotypes, is located in the intron of the *PSCA* gene and also overlapped with the *JRK* gene [56] (Figure S8). The A allele at rs2976391 is reported to be involved in promotor activity and transcription activity [56] (Tables S7 and S8). Although rs2978983 (A/G) currently has no reported biological function, the C-A subhaplotype may be associated with gene expression function and cause functional variation within a population. In the future, it would be interesting to examine the alteration of *PSCA* gene expression regarding the C haplotype including both subhaplotypes.

We detected C-A and A-G subhaplotypes that are maintained in both JPT and CHB. These two subhaplotypes have also been maintained in four metapopulations (non JPT/CHB EAS, SAS, EUR, and AFR). These haplotypes existing in the extant AFR further supported our estimated divergence time between the two subhaplotypes of ~240,000 ya. We detected the positive selection of subhaplotypes in these populations (Table S3). The C-A subhaplotype showed a signal of ongoing positive selection in the JPT, CHB, non JPT/CHB EAS, and SAS populations. In contrast, the A-G subhaplotype showed signals of ongoing positive selection in the CHB and AFR populations. Neither subhaplotype in EUR showed any signals. Unlike the case of CHB, non JPT/CHB EAS had a weaker signal on the A-G subhaplotype. The A-G subhaplotype is composed of derived alleles (A at rs2978391 and G at rs2978983), suggesting that it is younger than the C-A subhaplotype. Although the C-A subhaplotype is an ancestral subhaplotype, the extent of IAV was lower than the A-G subhaplotype in non-AFR populations (Table S3). This suggests that the signal of positive selection is stronger on the C-A subhaplotype than the A-G subhaplotype. Our study of the C haplotype in JPT identified two main findings. First, the C-A subhaplotype shared ancestral alleles with the T haplotype and it was difficult to detect unique derived SNPs (which are possible targets for the haplotype) in the C-A subhaplotype using a 2D SFS approach, suggesting that the combination of the C-A subhaplotype with the C allele at rs2249008 is a potential target of positive selection and would have a biologically important function, when compared to the C allele alone. Second, the A-G subhaplotype frequency is very similar to that of the C-A subhaplotype and this masks the signal of unique derived alleles in the C-A subhaplotype. Interestingly, the A-G subhaplotype showed a stronger signal than the C-A subhaplotype only in AFR. These findings suggest that the target of positive selection may have changed or reactivated during human population history. The status of positive selection may not have been necessarily inherited from an ancestor to the offspring populations (Figure S9). The lower limit of the beginning of positive selection was estimated as circa 30,000 to 11,000 ya (C-A subhaplotype) or circa 35,000 to 21,000 ya (A-G subhaplotype) among several metapopulations under positive selection. It is notable that the lower limit of the beginning of positive selection was similar among global populations, even though these populations have been isolated from each other for a long period and continued to differentiate. This further supports the idea that the positive selection target changed or was reactivated. 

In the forward simulation performed, we examined whether the *F*_ST_ value in each simulation was equal to or greater than that of rs2294408 (0.2547). The allele frequency trajectory revealed that high T allele frequency in the simulated JPT and large *F*_ST_ value required high T allele frequency in the ancestral population before admixture both under the neutral state and under positive selection on CHB lineages. This high T allele frequency implied that the Jomon people were likely to have had a high frequency of the T allele. In fact, in the extant Ryukyuan and Ainu people, who have a higher Jomon genetic component than the mainland Japanese [45], the frequency of the T allele on rs2294008, or its tightly linked adjacent allele, was higher than that in JPT (Ryukyuan: 0.701 (rs2294008) [57], Ainu: 0.975 (rs2976396, tightly linked with the rs2294008 T allele) [58]). To examine the relationship between the extant JPT and Jomon haplotypes, we checked the phylogenetic position of the Jomon haplotypes Ikawazu (IK002) and Funadomari (FUN5 and FUN23). Two of these Jomon samples (IK002 and FUN23) are closely related to the extant JPT-specific T haplotypes. FUN5 is also closely related to either T haplotypes that are extant JPT-specific or those widely shared between JPT and CHB. These observations support the possibility that the Jomon people had high T allele frequency and that the T allele in some extant Japanese haplotypes is derived from the Jomon haplotype.

Based on our analyses, we reconstructed the trajectory of the C and T haplotypes at rs2294008 along the Japanese population history (Figure S9). Positive selection on both C-A and A-G subhaplotypes began in the common ancestor of East Asians (including the ancestor of the Jomon). The ancestor of the Jomon diverged from ancestral populations in the East Asian continent from 15,000 to 38,000 ya [44,45,46], and migrated to the Japanese archipelago. Positive selection on the A-G subhaplotype then ceased or relaxed within the Jomon at some point, and this elevated the haplotype frequency containing T at rs2294008 within the Jomon. In contrast, the C allele frequency in the population was elevated in the ancestor of East Asian populations due to positive selection on the C haplotype. The ancestor of immigrant Yayoi farmers (who were genetically close to the extant Koreans) diverged from an ancestor of extant East Asian populations between 3000 and 3600 ya [59]. Immigrant Yayoi farmers migrated to the Japanese archipelago between 2500 and 3000 ya [42,60] and admixed with the Jomon who had high T allele frequency. Thus, although positive selection on both C-A and A-G subhaplotypes occurred in the ancestor of populations in the East Asian lineage, positive selection acted on only the C-A subhaplotype in the extant Japanese, and subsequently a high T allele frequency was observed in the Japanese. In future work, we will examine this scenario in more detail including Korean samples genetically close to the Japanese.

Although we cannot conclude whether selection on the A-G subhaplotype in the Japanese was relaxed or completely stopped, our study clearly showed that selection processes acting on rs2294008 differ between genetically close populations, such as JPT and CHB. This difference may be extended to other East Asian populations, SAS, EUR, and AFR. The relaxation or cessation of positive selection on the A-G subhaplotype may be a unique trait of the Japanese among the studied East Asian populations.

The associations between biological functions and rs2294008 and its adjacent region are complex, and interpreting the impact on the fitness of the T and C allele is difficult. For example, the T allele is reported as the risk allele of DGC, but the C allele (non-risk allele against DGC) is also reported as a risk allele for duodenal ulcers in Japanese [13] and Caucasian populations [12] based on genome-wide association studies. Thus, the difference between the haplotypes in biological function as well as selection modes/coefficients may have resulted in the functional variation of this region, which includes rs2294008, among genetically close East Asian populations. 

The above discussion is summarized as follows:

(i) Selection operated on the C allele (the non-risk allele) in the common ancestor of the Han Chinese and the Jomon people. The mode of positive selection in the Japanese is complex; selection on the A-G subhaplotype ceased or relaxed at some point along the Japanese lineage, but ongoing selection occurred on the C-A suphaplotype. Relaxation or cessation of positive selection on the A-G subhaplotype may have led to low frequency of the C allele in the extant JPT.
(ii) The ancestral population (the Jomon people) had a high T allele frequency, which led to a high T allele frequency in the extant Japanese, even though the Jomon people experienced admixture with immigrant Yayoi farmers. These factors result in the large T/C allele frequency difference between JPT and CHB.

This study is the first to report the complex positive selection modes acting at rs2294008 among human populations, even between JPT and their genetically closest population. Our findings support that positive selection targets can change during human history over relatively short periods.

## Figures and Tables

**Table 1:** Summary statistics of two-dimensional site frequency spectrum (2D SFS).

Population	JPT		CHB	
Core Region	143755915- 143770914		143755876- 143771875	
*n* ^#^	208 (C = 77, T = 131)		206 (C = 155, T = 51)	
*S* ^†^	91		88	
Tested Allele	C	T	C	T
Allele Frequency	0.370	0.630	0.752	0.248
F_c_^§^	0.167 (0.834)	0.833 (>0.999)	0.352 × 10^−1^ (0.223 × 10^−2^) **	0.869 (>0.999)
G_c0_	9.60 (0.693)	31.84 (0.975)	1.84 (0.167 × 10^−2^) **	25.13 (>0.999)
L_c0_	0.178 × 10^−1^ (0.708)	0.259 (>0.999)	0.565 × 10^−2^ (>0.167 × 10^−2^) **	0.130 (>0.999)
G^*^_c0_	22.50	46.23	5.00	30.69
γ*(10)	0.500	0.700	0.000	0.962
*i*_max_	40	130	8	50
i^*^_max_	0	29	75	21

^§^ *q*-value in parentheses represents under null model. ** represents *q*-value < 0.01. ^#^ *n* represents the number of samples. ^†^ *S* represents the number of segregating sites in the tested population.

## Supplementary Materials

The following are available online at https://www.mdpi.com/2073-4425/11/7/775/s1. Figure S1: Demographic model of the Jomon (JMN), an ancestral population inhabiting the Asian continent (A_CNT), simulated Japanese in Tokyo (simulated JPT) and simulated Han Chinese in Beijing (simulated CHB); Figure S2: *F*_ST_ distribution per 21.9 kb windows; Figure S3: SNPs with high *F*_ST_ as core for EHH; Figure S4: Distribution of PBS values and position of rs2294008 for JPT (**a**) and CHB (**b**) based on 9,051,837 genome-wide PBS values; Figure S5: Trajectories of T allele frequency at SNPs with high T allele frequency and high *F*_ST_ under neutral state in the CHB lineage; Figure S6: Trajectories of T allele frequency at SNPs with high T allele frequency and high *F*_ST_ under positive selection on the C allele in the JPT lineage; Figure S7: The decay of *r*^2^ with rs2294008; Figure S8: Genomic context of rs2976391; Figure S9: Positive selection status among global populations; Table S1: T allele frequency and *F*_ST_ values at rs2294008 in 1KGP populations; Table S2: List of *F*_ST_ of top 50 SNPs; Table S3: 2D SFS analysis of two subhaplotypes in global populations; Table S4: Differentiation level classified by allele frequencies of the last generation of JMN and A_CNT; Table S5: List of extant JPT and CHB haplotypes in 21.9 kb LD block; Table S6: Nucleotide diversity at LD block and flanking 100 kb regions; Table S7: Genes and transcript consequences of rs2976391; Table S8: Motif feature consequences of rs2976391.
